# Decoding the Role of Lipid Metabolism and Membrane Dynamics in Melanoma

**DOI:** 10.3390/ijms27041715

**Published:** 2026-02-10

**Authors:** Maria Elena Pisanu, Egidio Iorio, Francesco Facchiano, Mattea Chirico, Maria Luisa Scattoni, Claudio Tabolacci

**Affiliations:** 1 Core Facilities, High Resolution NMR Unit, Istituto Superiore di Sanità, 00161 Rome, Italy; mariaelena.pisanu@iss.it (M.E.P.); egidio.iorio@iss.it (E.I.); mattea.chirico@iss.it (M.C.); 2 Department of Oncology and Molecular Medicine, Istituto Superiore di Sanità, 00161 Rome, Italy; francesco.facchiano@iss.it; 3 Coordination and Promotion of Research, Istituto Superiore di Sanità, Viale Regina Elena 299, 00161 Rome, Italy; marialuisa.scattoni@iss.it

**Keywords:** melanoma, lipid metabolism, membrane, UV radiation, diet

## Abstract

Cutaneous melanoma is a highly aggressive type of cancer with a poor prognosis at advanced stages. Accumulating evidence demonstrates that metabolic reprogramming is essential for melanoma, allowing it to adapt to both cellular changes, due to its genetic instability, and to micro-environmental stimuli. This review provides an overview of how melanoma cells remodel membrane lipids during melanoma progression with a focus on how environmental stresses (e.g., UV radiation) affect tumor aggressiveness and therapy resistance by reshaping membrane structure, fluidity, and composition. Dietary lipids, especially omega-3 polyunsaturated fatty acids (PUFAs), further modulate membrane properties and can sensitize melanoma cells to oxidative stress and ferroptosis, revealing potential therapeutic vulnerabilities. Finally, we discuss emerging evidence that lipid signatures, including circulating lipid profiles and melanoma-derived exosomes, have prognostic and predictive value. Together, these insights emphasize the importance of lipid metabolism and membrane architecture as key factors in melanoma biology and as promising targets for personalized interventions.

## 1. Introduction

Cutaneous melanoma (hereafter melanoma), a malignant neoplasm that arises from melanocytes (the melanin-producing cells in the basal layer of the epidermis), represents the most aggressive and lethal type of skin cancer. Although it represents a relatively rare malignancy, accounting for less than 2% of all cancer diagnoses worldwide, it is responsible for approximately 80% of skin cancer-related deaths [1]. The incidence of melanoma has continued to increase in recent decades particularly among fair-skinned populations in Western countries, where exposure to ultraviolet (UV) radiation represents the principal environmental risk factor [2,3]. Moreover, this type of cancer is generally more frequent in elderly males, showing not only a distinct sex-related variation in tumor distribution but also an age-dependent risk [4]. Genetic predisposition, along with pigmentation characteristics and patterns of UV exposure, strongly influence individual susceptibility. In fact, approximately 5–10% of melanoma cases are familial [3]. *CDK4* (cyclin-dependent kinase 4) and *CDKN2A* (cyclin-dependent kinase inhibitor 2A) are the most well-established high-penetrance genes associated with familial melanoma. In particular, mutations in these genes disrupt the p16^INK4a^-Rb tumor suppressor pathway, allowing melanocytes to survive to DNA damage from UV radiation and progress toward malignancy [3,5]. Moreover, genome-wide association studies (GWASs) have identified numerous additional *loci* that influence melanoma risk, including *TYR* (tyrosinase), *PLA2G6* (phospholipase A2 group VI), *TERT* (telomerase reverse transcriptase), *ATM* (ATM serine/threonine kinase), and *TP53* (p53), among others [6]. Melanocortin 1 receptor (*MC1R*) gene variants are also associated with an increased risk for melanoma [7]. Indeed, MC1R plays a crucial role in pigmentation, as it regulates melanin production in melanocytes by shifting the synthesis from pheomelanin to eumelanin, which represent the two main types of melanin that differ in color and photoprotective capacity [8]. Melanoma is characterized by a high mutational burden with somatic oncogenic mutations in *BRAF* and *NRAS* being the most frequent. These mutations activate the mitogen-activated protein kinase (MAPK) pathway, leading to uncontrolled cellular growth [9]. Interestingly, *MC1R* variants may affect the frequency of several somatic mutations, including *BRAF*, *NRAS*, and *TERT* [10].

Highly aggressive features and strong metastatic potential characterize melanoma, which largely explains its high mortality compared to other skin cancers [11]. Surgical excision is the standard for localized tumors, while the identification of key driver mutations in *BRAF*, *NRAS*, and *TERT* has led to the development of target therapies. In particular, inhibitors targeting BRAF(V600) mutations (BRAFi) in combination with MEK inhibitors (MEKi) have represented a significant improvement in the treatment of melanoma [9]. Nevertheless, acquired resistance to these treatments invariably develops, highlighting the activation of genetic and epigenetic mechanisms that activate alternative signaling pathways. Moreover, immunotherapy, including immune checkpoint inhibitors targeting PD-1 or CTLA-4, has become a significant progress in melanoma treatment [12]. To date, combination therapies represent the first-line options for advanced forms [13,14]. Melanoma is characterized by high heterogeneity and remarkable metabolic plasticity (e.g., switching between glycolysis and oxidative phosphorylation) that allows tumor cells to adapt to external stimuli, supporting survival, metastasis, and resistance to therapies [15]. Among the key metabolic pathways reprogrammed in melanoma, lipid metabolism has emerged as a key regulator of melanoma progression and behavior [16].

Lipids are multifunctional molecules that play a critical role as a source of energy, structural components of biological membranes, and signaling molecules. It is well established that the dysregulation of some lipid metabolic enzymes such as fatty acid synthase (FASN), stearoyl-CoA desaturase 1 (SCD1), and acetyl-CoA carboxylase (ACC) promotes de novo lipogenesis, generating unsaturated fatty acids and complex lipid species that enhance membrane fluidity and receptor clustering [17]. Indeed, in several cancers (including melanoma), alterations in the composition and organization of membrane lipids regulate not only receptor clustering and signaling but also metabolic adaptation. Lipid rafts and tumor-specific fatty acid profiles integrate these structural functions, highlighting how lipid remodeling is a key factor in melanoma aggressiveness and therapeutic resistance [18,19,20,21,22]. Moreover, the activation of sterol regulatory element-binding protein 1 (SREBP1), a trans-endoplasmic reticular membrane protein factor, as well as other SREBPs, represents a key regulator of fatty acids, cholesterol, and glucose metabolism [23]. Elevated SREBP activity drives the expression of FASN, SCD1, and ACC, among others with the consequent promotion of cell proliferation, invasiveness, and resistance to targeted therapies [24,25,26]. Aberrant lipid signaling has been shown to regulate several oncogenic cascades including the PI3K/AKT/mTOR, MAPK/ERK, and YAP/TAZ pathways [16,23,27,28]. Furthermore, lipid metabolism contributes to the pathogenetic mechanisms that enable melanoma cells to evade immune surveillance [15]. Therefore, this review, in addition to providing an overview of lipid nomenclature and biosynthetic pathways, focuses on the role of lipid metabolism in melanoma and its contribution to cancer progression with particular attention to the composition and dynamics of membrane lipids. We discuss how melanoma cells reprogram lipid metabolism to alter membrane fluidity and microdomain organization, facilitating rapid adaptation to environmental stresses. Finally, we provide an overview on how dietary polyunsaturated fatty acids (PUFAs) may influence the melanoma membrane and the possible role of circulating lipids and extracellular vesicles (EVs), including the signal molecules transported within them, as melanoma biomarkers.

## 2. Biosynthesis and Classification of Lipid Membrane Components

### 2.1. Structure, Distribution, and Biological Functions

Biological membranes are heterogeneous, amphipathic bilayers that act as selective barriers separating the intracellular and extracellular environments [29]. In eukaryotes, they also establish the compartmentalization of organelles and provide dynamic microdomains or lipid rafts and other specialized functional regions that are responsible for coordinating signaling, membrane trafficking, and receptor organization [29]. The diverse functions of these membranes are a consequence of the different chemical structures and biophysical properties of lipids as well as their distribution patterns. Cellular membranes are predominantly composed of phospholipids, sterols, and sphingolipids, which are each derived from fatty acids that function as essential precursors in lipid metabolism.

**Figure 1:**
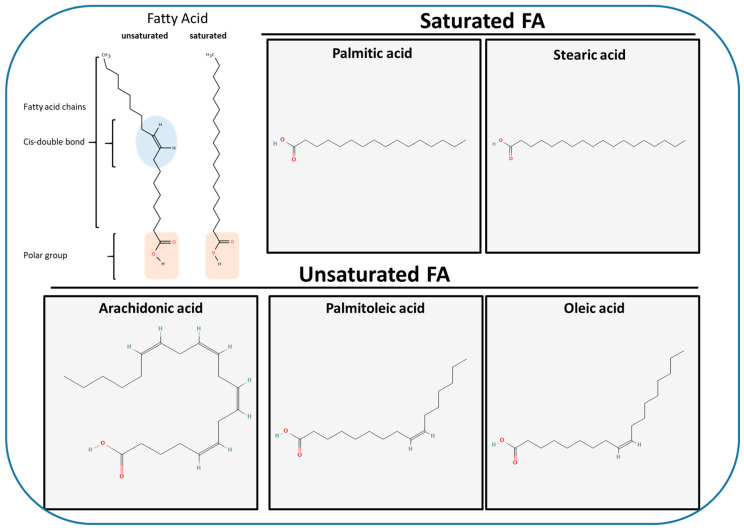
Chemical structure of fatty acids (FAs) and their degree of unsaturation of fatty acids (UFAs). FAs are carboxylic acids composed of a hydrocarbon chain of variable length terminating with a carboxyl group. They differ in chain length, degree of unsaturation, and position of double bonds, which together determine their physical properties and biological functions. Examples in the figure include (a) saturated fatty acids: palmitic acid (16:0), stearic acid (18:0) with no double bounds; (b) monounsaturated fatty acids (MUFAs): palmitoleic acid (16:1) and oleic acid (18:1) with each FA containing one double bond; and (c) polyunsaturated fatty acids (PUFAs) with two or more double bonds: arachidonic acid (20:4).

Fatty acids (FAs), which can be classified into saturated fatty acids (SFAs), monounsaturated fatty acids (MUFAs), and polyunsaturated fatty acids (PUFAs), are composed of a terminal carboxyl group linked to a hydrocarbon chain of varying lengths and degrees of unsaturation (Figure 1).

**Figure 2:**
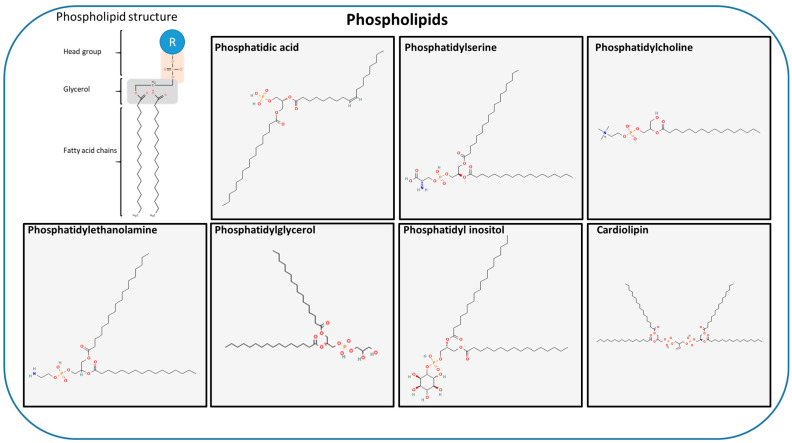
Chemical structure of phospholipids (PLs). Phospholipids are characterized by a glycerol backbone esterified with fatty acids at the sn-1 and sn-2 positions and by a polar head group (R) composed of a phosphate and an alcohol (e.g., serine, choline, ethanolamine, glycerol, inositol). Cardiolipin is a phospholipid in which two phosphatidylglycerol units are linked, resulting in four acyl chains.

They play a crucial role in diverse cellular processes, including the formation of membrane phospholipids. In particular, MUFAs and PUFAs regulate permeability and fluidity properties, acting in an opposite manner to SFAs, which instead create the rigidity and a gel-like state [30,31]. In eukaryotes, phospholipids constitute the main class of structural lipid, accounting for 50–60% of the overall membrane lipid content. They share a common glycerol phosphate backbone but differ in their headgroups and the composition of their fatty acyl chains, which can vary in length, saturation and the position of double bonds [32]. These molecular differences strongly influence the membrane fluidity, curvature and overall organization. Examples include phosphatidic acid (PA), phosphatidylglycerol (PG), phosphatidylcholine (PC), phosphatidylethanolamine (PE), phosphatidylserine (PS) and phosphatidylinositol (PI) as well as their phosphorylated derivatives (PIPs) (Figure 2) [32,33].

It is noteworthy that poorly metastatic melanoma cells have been shown to contain more saturated and shorter fatty acid tails when compared to highly metastatic melanoma cells [34].

Phosphatidic acid (PA) is the simplest negatively charged phospholipid and is typically found in membranes at low concentrations (~5%) due to its rapid conversion by lipid phosphate phosphohydrolases [35,36]. Structurally, PA comprises a phosphate group attached to the sn-3 position of glycerol with two fatty acyl chains linked to the remaining sn-positions. PA plays a critical role in various signaling pathways and is involved in phospholipid and glycerolipid metabolism, neuroendocrine exocytosis, protein kinase regulation, small G-protein activation and membrane fusion and fission [37,38,39,40,41]. Phosphatidylglycerol (PG) is derived from the esterification of glycerol with a phosphate group. Its structure includes a backbone of glycerol linked to two fatty acid chains and a phosphoglycerol headgroup that retains two hydroxyl groups [42]. Phosphatidylcholine (PC) is the most abundant class of phospholipids in cellular membranes, accounting for around 40–50% of total cellular phospholipids. However, concentrations and subcellular distributions can vary between different cells [43]. This predominance is crucial in maintaining membrane integrity and functionality [44]. Phosphatidylcholine primarily exists as diacyl-PC, which comprises two ester-linked fatty acids. Lesser forms, such as ether-linked alkyl-PC and alkenyl-PC (choline plasmalogens -vinyl ether bond), exhibit different structural properties [45,46,47]. Beyond its structural functions, PC acts as a precursor for sphingomyelin (SM) and other lipid species, including PA, lysophosphatidylcholines (LysoPCs), PS, and platelet-activating factor (PAF), thus contributing to cell signaling, regulating lipoprotein composition in circulation, and facilitating membrane trafficking [48]. Phosphatidylethanolamine (PE) is the second most abundant phospholipid in mammalian membranes, accounting for 15–25% of the total phospholipids [49]. It is particularly enriched in mitochondrial membranes and is confined to the cytosolic leaflet of the plasma membrane [48]. The small ethanolamine headgroup imparts distinct chemical and biophysical properties to PE, allowing it to adopt a non-bilayer structure [49]. PE significantly influences membrane architecture, rigidity, fluidity, and permeability, and it is involved in key cellular processes such as membrane fusion, diacylglycerol (DAG) production via phospholipase C (PLC), and protein modifications mediated by reactive aldehydes [48]. Phosphatidylserine (PS) is a minor phospholipid in mammalian cells, accounting for 2–15% of total phospholipids with particularly high levels found in the cerebral cortex [50]. It is also prevalent in organelle membranes, such as those of the mitochondria and ER, where it acts as a substrate for PE synthesis [48]. Structurally, PS exhibits a negatively charged serine headgroup that reacts strongly with divalent cations. Unlike PC and PE, PS is exclusively found as a diacyl species containing an unsaturated fatty acid at the sn-2 position [50]. PS is crucial for membrane integrity, fluidity, and permeability. It functions as a precursor for PE and interacts with sphingolipids and cholesterol to influence the membrane structure. PS also participates in various signaling pathways, including those related to apoptosis and immune regulation [51,52]. Phosphatidylinositol (PI) is an anionic phospholipid characterized by a myo-inositol headgroup. In eukaryotic cells, the predominant phosphoinositide species are monophosphoinositide (PI) and diphosphoinositide (PI4P and PI5P), accounting for up to 10% of total cellular phospholipids. Phosphatidylinositol (PI) is widely distributed across cellular membranes, including the ER and Golgi apparatus [53,54]. As the simplest member of inositol-containing phospholipids (phosphoinositides), PI acts as a precursor to several phosphorylated derivatives. PI participates in cellular signaling, generating DAG through PLC and releasing fatty acids via PLA2. It contributes to inflammation and immune regulation, and it facilitates the membrane anchoring of glycosyl–phosphatidylinositol (GPI)-anchored proteins [55]. Despite existing in low concentrations, phosphorylated derivatives play key roles in membrane organization and the regulation of protein activity.

Cardiolipin (CL), also known as diphosphatidylglycerol (DPG), is a distinctive tetra-acylated phospholipid that is predominately localized in the inner and outer mitochondrial membranes [56]. CL constitutes ~25% of total mitochondrial polar lipids and is fundamental to mitochondrial function [57,58]. It contributes to the organization of the respiratory chain and interacts with ADP/ATP and complex III and IV proteins [59,60,61]. It also plays a role in mitochondrial fission and fusion processes, and it controls the release of apoptotic factors [62,63,64].

**Figure 3:**
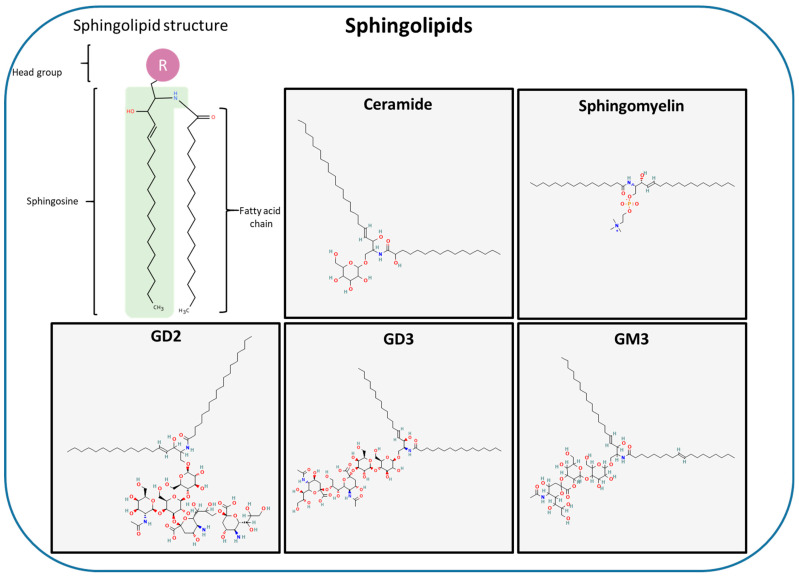
Chemical structure of sphingolipids (SLs). Sphingolipids consist of a sphingoid base (e.g., sphingosine), an N-acyl chain forming ceramide, and a variable head group. The figure shows some SLs as ceramide, sphingomyelin, and complex glycosphingolipids (e.g., GD2, GD3, GM3). The headgroups—hydroxyl, phosphocholine, or oligosaccharides—define ceramide, sphingomyelin, and gangliosides, respectively. GD2, GD3, and GM3 differ in their oligosaccharide composition.

Sphingolipids (SLs) (Figure 3) represent a distinct class of membrane lipids characterized by a different backbone that includes variations in hydroxylation and unsaturation as well as an N-acyl chain and a headgroup. They encompass ceramide (Cer), SM and glycosphingolipids [65].

Ceramide (Cer), a non-bilayer-forming lipid, is composed of a sphingosine (Sph) backbone linked to a single acyl chain via an amide bond. It lacks a distinct headgroup and displays pronounced hydrophobicity [66]. Ceramides isoforms exhibit tissue-specific expression, generating Cer distinct species with varying acyl-chain lengths that differentially influence cellular signaling. For instance, C16-Cer is linked to apoptosis, whereas C18-Cer is associated with growth arrest [67]. Ceramides can form highly hydrophobic, gel-like domains in membranes that act as platforms for protein–lipid interactions and participate in lipid raft formation [68,69]. Phosphosphingolipids are characterized by a polar headgroup—typically choline, ethanolamine, or glycerol. This gives them hydrogen bond donor and acceptor capabilities. This class includes ceramide-1-phosphate (Cer-1-P), dihydroceramide-1-phosphate (D-Cer-1-P), ceramide phosphoethanolamines (Cer-PE), sphingomyelin (SM or Cer-1-PC), dihydrosphingomyelin, and lyso-PSLs. Sphingomyelin (SM) is a major SL in animal plasma membranes, accounting for ~15% of cerebral lipids. It is characterized by an enrichment in saturated acyl chains and participates in the signaling, proliferation, apoptosis, and uptake of cholesteryl ester. It also contributes to lipid raft formation by interacting with cholesterol [68,69]. SM preferentially localizes to the outer leaflet of membranes but is also present in nucleus, mitochondria, and liver chromatin [70,71,72]. Sphingolipids constitute approximately 10–20% of total cellular lipids. Despite their relatively low abundance, sphingolipids play significant roles in cellular signaling, membrane organization, and interactions between cells and their environments, acting as either second messengers or extracellular ligands for receptors. Glycosphingolipids (GSLs), derived from Cer, replace the phosphoryl headgroup with mono-, di- or oligosaccharides. Gangliosides (GM3, GD2 and GD3) are a subclass of GSLs that contain terminal sialic acid. GSLs preferentially localize to lipid rafts, where they modulate cell signaling, adhesion, and communication [73]. They also act as antigens, influencing immune cell responses and growth factor receptor interactions [73].

**Figure 4:**
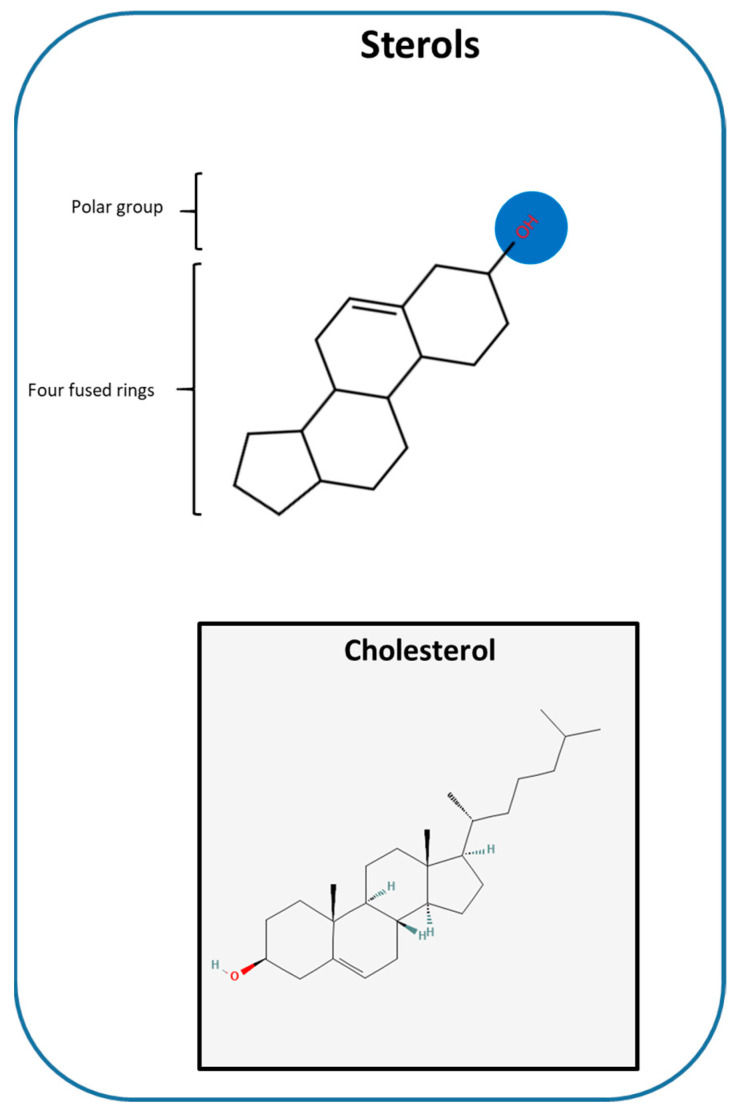
Chemical structure of sterols. Cholesterol is the major sterol in mammalian membranes, which is characterized by its rigid four-ring structure and a hydrophilic hydroxyl group.

Sterols (Figure 4), mainly represented by cholesterol in the mammalian cell membranes, are amphipathic lipids characterized by a rigid tetracyclic structure, a flexible aliphatic side chain and a polar hydroxyl group that interacts with the headgroups of phospholipids [74,75].

Within membranes, cholesterol is asymmetrically distributed with 20–50% localized in the cytosolic leaflet of the plasma membrane and less than 15% in the endoplasmic reticulum (ER), where it modulates membrane fluidity, rigidity, and permeability [76,77]. Beyond its structural roles, cholesterol is crucial for signaling through its interactions with proteins and receptors. It also acts as a precursor for bile acids and steroid hormones [78,79]. Cholesteryl esters are important for the transport of fatty acids via lipoproteins, and, together with sphingolipids, they promote the formation of microdomains, which are commonly referred to as lipid rafts [68,69].

### 2.2. Lipid Biosynthesis and Turnover

**Figure 5:**
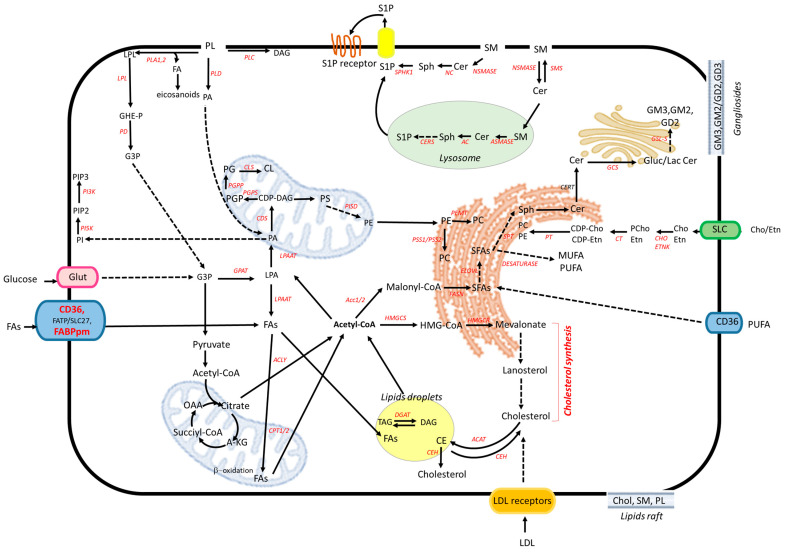
Schematic representation of lipid metabolic network involved in membrane biogenesis and degradation. Metabolites: CDP-Cho/Etn, cytidine diphosphate choline/ethanolamine; CDP-DAG, cytidine diphosphate diacylglycerol; CE, cholesteryl ester; Cer, ceramide; Cho/Etn choline/Ethanolamine; CL, cardiolipin; DAG, diacylglycerol; FA, fatty acid; FAs, fatty acids; G3P, glycerol-3-phosphate; GHE-P, glycerol-phospho-head group; Gluc/Lac Cer, glucosyl/lactosyl ceramide; GM3, GD2, GM2 gangliosides; HMG-CoA, 3-hydroxy-3-methylglutaryl-coenzyme A; LPA, lysophosphatidic acid; LPL, lysophospholipid; MUFA, monounsaturated fatty acid; OAA, oxaloacetate; PA, phosphatidic acid; PC/PE, phosphatidylcholine/phosphatidyletnanolamine; PCho/PEtn phosphocholine/phosphoethanolamine; PG, phosphatidylglycerol; PGP, phosphatidylglycerophosphate; PI, phosphatidylinositol; PIP2, phosphatidylinositol diphosphate; PIP3, phosphatidylinositol triphosphate; PL, phospholipid; PL, phospholipid; PS, phosphatidylserine; PUFA, polyunsaturated fatty acid; S1P, sphongosine-1-phosphate; SFAs, saturated fatty acids; SM, sphingomyelin; Sph, sphingosine; TAG, triacylglycerol; α-KG, α-ketoglutarate. Enzymes and transporters: (i) FA metabolism. ACC1/2, acetyl-CoA carboxylase; ACLY, ATP citrate lyase; FASN, fatty acid synthase; ELONVL elongase; desaturase; DGAT, acyl CoA:diacylglycerol acyltransferase; CD36; FATP/SLC27, fatty acid transport protein/solute Carrier 27; FABPpm, plasma membrane fatty acid binding protein, cpt1/2, carnitine O-palmitoyltransferase 1/2; (ii) PL metabolism. CHOK/ETNK, choline/ethanolamine kinases; CT, cytidyltransferase; PT, phosphocholine/phosphoethanolamine transferase; PSS1/PSS2, phosphatidylserine synthases 1 and 2; PLC, phospholipase C; PLD, phospholipase D; Pla1, phospholipase A1; Pla2, phospholipase A2; LPL, lysophospholipase; PD, glycerophosphodiesterase; PI5K, phosphatidylinositol-5-kinase; PI3K, phosphatidylinositol-3-kinase; PEMPT, phosphatidylethanolamine N-methyltransferase; LPAAT, lysophosphatidic acid acyltransferase; CDS, cytidine diphosphate diacylglycerol synthase; PGPS, phosphatidylglycerophosphate synthase; PGPP, phosphatidylglycerophosphate phosphatase; CLS, cardiolipin synthase; PISD, phosphatidylserine decarboxylase proenzyme; SLC transporters; (iii) Cholesterol metabolism. HMGCS, HMG-CoA synthase; HMGCR, HMG-CoA reductase; ACAT, acyl-coenzyme A (CoA):cholesterol acyltransferases; CEH, cholesteryl esters hydrolase; LDL receptor, LDL (low-density lipoprotein) receptor; (iv) SL metabolism. ASMASE, acid sphingomyelinase; AC, acid ceramidase; CERS, ceramide synthase; SMS, sphingomyelin synthase, NSMASE, neutral sphingomyelinase; NC, acid ceramidase; SPHK1, sphingomyelin kinase1; GCS, glucosylceramide synthase; GSL-s synthase, ganglioside synthase; cert, ceramide transport protein; S1P receptor, sphingosine phosphate receptor.

The ER, mitochondria, and the Golgi apparatus play a key role in the biosynthesis of various lipid classes of cellular membranes. In contrast, lipid hydrolysis primarily occurs within the lysosomal compartment, particularly in intralysosomal luminal vesicles where multiple hydrolases are active. Lipids are transported to lysosomes via endocytosis and autophagy pathways. The maintenance of cellular lipid homeostasis is due to a coordinated interplay between fatty acid (FA) uptake, de novo lipogenesis, intracellular storage, and oxidative degradation (see the schematic network of lipid metabolism in Figure 5).

Fatty acids can be synthesized de novo within the cytosol or acquired by transporters such as CD36, FATP/SLC27 (fatty acid transport protein/solute carrier family 27) family members, and FABPpm via uptake from the extracellular environment. Once inside the cells, fatty acids are converted to acetyl-CoA, which directs them towards lipid synthesis, mitochondrial β-oxidation, or storage in lipid droplets [80]. De novo lipogenesis is primarily active in hepatocytes and adipocytes, where it functions as an endogenous source of FAs. Acetyl-CoA, which is derived from substrates such as glucose, is converted into malonyl-CoA. This reaction is catalyzed by acetyl-CoA carboxylases (ACC1/2; encoded by *ACACA* and *ACACB*) and acts as a rate-limiting step. Fatty acid synthase (FASN) then catalyzes the condensation of acetyl-CoA with malonyl-CoA to produce long-chain SFAs, such as stearoyl-CoA (18:0) and palmitoyl-CoA (16:0), which are the most common saturated fatty acid [81,82,83]. These fatty acids can subsequently be elongated by elongases (ELOVLs) or desaturated by stearoyl-CoA desaturases (SCD1/5) and fatty acid desaturase (FADs), yielding a diversity of FAs, including MUFA oleic acid (18:1) and palmitoleic acid (16:1), and the PUFA arachidonic acid (ARA, 20:4 n-6) [84]. MUFAs can be obtained entirely through an endogenous process, whereas PUFAs, such as omega-6 and omega-3, are essential components of eukaryotic membranes but cannot be synthesized de novo. Their precursors, linoleic acid and α-linolenic acid, must therefore be obtained from the diet. Following cellular uptake, these essential fatty acids undergo a series of elongation and desaturation reactions to generate long-chain PUFAs, such as omega-6 di-homo-gamma linolenic acid (DGLA) and ARA and omega-3 eicosapentaenoic acid (EPA) and docosahexaenoic acid (DHA) [30,85]. These are then incorporated into the membrane phospholipids and TAGs (neutral lipids composed of three fatty acids esterified to a glycerol backbone) stored in lipid droplets.

Phosphatidic acid (PA) is a central intermediate in lipid metabolism and is synthesized from glycerol-3-phosphate via sequential reactions of acylation. PA can be dephosphorylated into DAG and TAG with the interconversion between PA and DAG regulated by DAG kinase and PA phosphatases [41]. Glycerophospholipids, such as PC and PS, are derived from PA downstream. PC is synthesized in the ER through the Kennedy pathway with cytidylyltransferase (CT) acting as the rate-limiting enzyme [86]. PS, in mammalian cells, is synthesized in mitochondrial-associated membranes (MAMs) and the ER through calcium-dependent base-exchange reactions catalyzed by PS synthases (PSS1 and PSS2), employing PC or PE as precursors [87]. Under normal conditions, PS primary localizes in the cytosolic leaflet of the plasma membrane but can translocate to the outer leaflet during processes such as apoptosis, where it acts as a phagocytic signal [88]. Phosphatidylethanolamine (PE) is produced either via the CDP–ethanolamine pathway or through the mitochondrial decarboxylation of PS [86,89]. The synthesis of phosphoinositides, which are crucial for signaling and membrane identity, arises from PA via CDP–DAG synthase and myo-inositol phosphatidyltransferase [90,91]. Mitochondria synthesize PG from imported PA, which is then converted into cardiolipin (CL) by cardiolipin synthase [92]. CL is a critical phospholipid for the functionality of the inner mitochondrial membrane.

Sphingolipid biosynthesis begins in the ER with serine palmitoyltransferase (SPT), which uses palmitoyl-Coa to produce 3-ketosphinganine. This is then reduced and acylated to form ceramide. Ceramide, a pivotal sphingolipid intermediate, can be also produced through SM hydrolysis and salvage pathways involving the reacylation of Sph [93,94]. Sphingomyelin (SM) can also be produced by the acylation of lyso-SM or the transfer of phosphocholine to ceramide [95]. Ceramide is then trafficked to the Golgi either via vesicular routes or non-vesicular transfer by the ceramide transfer protein CERT for conversion into SM and complex glycosphingolipids [96,97,98]. Glycosphingolipids assembly in the Golgi occurs by sequential glycosyl- and sialyltransferase reactions that are spatially organized along the cis–trans Golgi axis. The order and localization of these enzymes determine oligosaccharide structures. Golgi architecture and lipid microenvironments thus control the diversity and functional roles of glycosphingolipids, which are critical for membrane domain formation and cell-surface recognition.

Cholesterol is synthesized from acetyl-CoA, which is transformed into 3-hydroxy-3 methylglutaryl-CoA (HMG-CoA). This is then reduced to mevalonate via the mevalonate pathway (HMG-CoA reductase acting as the rate-limiting step) in the ER/cytosol and is subsequently distributed to other compartments. Mevalonate is then converted into squalene, which is used in cholesterol synthesis.

Crosstalk between cholesterol and sphingolipid metabolism creates regulatory axes that stabilize membrane biophysical properties [99]. Cholesterol homeostasis is maintained through the synthesis, uptake (via the LDL receptor (LDLR)/Apo-B100 pathway) and active transport via lipid transfer proteins (e.g., OSBP/ORP family, STARD proteins) and membrane contact sites that link the ER, the Golgi apparatus, endosomes and the plasma membrane. These processes are tightly regulated by negative feedback mechanisms that sense intracellular cholesterol levels [99,100].

In addition to synthesis and uptake, lipid homeostasis is tightly regulated through lipid storage and lipid remodeling. Lipid droplets (LDs) are dynamic intracellular organelles composed of a core of neutral lipid, mainly TAGs and cholesteryl esters, which are surrounded by a monolayer of phospholipids and proteins that store neutral lipids [80,101,102,103]. Lipid droplets not only act as reservoirs of metabolic intermediates but also as a buffer of excess or toxic lipids, thereby preventing lipotoxicity and oxidative stress. The remodeling of phospholipid distribution involves multiple interconnected mechanisms including acyl-chain remodeling via the Lands cycle and head-group interconversions, such as the conversion of PE to PC, glycerophospholipids deacylation and hydrolysis [104,105]. These processes are mediated by the coordinated action of PLA2, PLA1 and lysophospholipase as well as PLC and PLD. These mechanisms generate a range of signaling lipids, including DAG, PA, lysophospholipids, free fatty acids, PAF, ARA and its derivates (eicosanoids), which can act as second messengers and modulate metabolic flux, or be reintegrated into biosynthetic pathways or funneled into β-oxidation for energy production [16,106]. In parallel, sphingolipids remodeling constitutes another important degradative and regulatory pathway. Lysosomal sphingomyelinases and ceramidases catalyze the breakdown of sphingolipids into sphingosylphosphorylcholine (SPC), Sph, sphingosine-1-phosphate (S1P), ceramide-1-phosphate (C1P) and Cer, which participate in key signaling cascades and membrane homeostasis [107].

Fatty acid β-oxidation is another catabolic pathway that provides energy, particularly under nutrient-limited conditions or oxidative stress. Carnitine palmitoyltransferase I (CPT1) catalyzes the conversion of fatty acyl-CoAs into acylcarnitines, which are transported into the mitochondria, for β-oxidation and ATP production [30]. Finally, lipid catabolism can also lead to lipid peroxidation by reactive oxygen species (ROS), particularly in the case of PUFAs, which can significantly alter the physical properties of cellular membranes [108] or result in the formation of reactive compounds that cross-link DNA or proteins, thereby exerting further toxic effects [108,109,110]. Extensive lipid peroxidation can ultimately trigger ferroptosis, which is a regulated form of iron-dependent, non-apoptotic cell death [110].

According to the above reported studies, the knowledge about lipid biosynthesis and turnover is increasing also due to the recent availability of novel mass spectrometry-based technologies [111]. On the other hand, lipid homeostasis is emerging in recent years as one of the most promising fields of study in order to develop new strategies against melanoma drug resistance, as recently demonstrated in a model of NRAS-mutant melanoma cells [112].

### 2.3. Membrane Microdomains and Extracellular Vesicles

**Figure 6:**
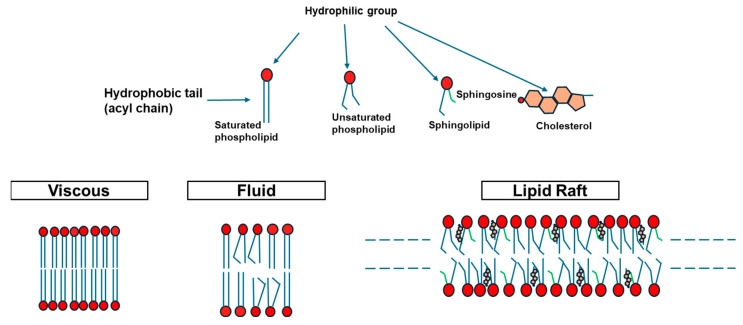
Schematic representation of biophysical properties of lipid bilayers. Upper panel shows the main lipids in cell membranes. Bottom panel indicates examples of the different lipid membrane phases and a schematic representation of lipid rafts.

Classical membrane biology was long guided by the fluid mosaic model [113], which depicted membranes as largely homogeneous, fluid bilayers in which proteins diffuse freely. By the late 1970s and 1980s, however, biochemical and biophysical studies revealed that membrane lipids do not mix ideally; instead, subsets of lipids can segregate laterally into distinct phases, introducing an additional level of structural organization beyond the basic bilayer. In particular, early evidence showed that membranes could be separated into detergent-resistant and non-resistant fractions, hinting at underlying heterogeneity. Subsequent biochemical and biophysical studies revealed that lipids can segregate into phases with distinct physical properties, leading to the concepts of liquid-ordered (Lo) and liquid-disordered (Ld) domains and ultimately to the membrane raft hypothesis (Figure 6).

The degree of saturation of fatty acyl chains, in particular, plays a key role in regulating membrane fluidity and phase behavior. Saturated lipids, characterized by straight acyl chains, pack tightly and favor the formation of more rigid membrane domains, whereas unsaturated lipids, which contain one or more cis double bonds, introduce kinks that reduce packing efficiency and increase membrane fluidity (Figure 6). These differences underlie the coexistence of distinct membrane phases, which are commonly described as Lo and Ld states. Lo domains are typically enriched in saturated lipids and cholesterol, resulting in a more ordered yet laterally mobile structure, while Ld regions are enriched in unsaturated phospholipids and display higher fluidity and disorder. The dynamic balance between these phases contributes to membrane heterogeneity and plays a critical role in regulating protein localization, signaling platforms, and intracellular trafficking. The length of degree of unsaturation of acyl chains sterols such as cholesterol play a fundamental role in tuning the membrane fluidity of the transition between Ld and Lo states [114]. Rafts were proposed as dynamic, cholesterol- and sphingolipid-enriched assemblies that generate locally ordered regions capable of recruiting specific lipids and proteins. These observations led to the lipid raft hypothesis [115,116].

In the 2000s, different studies described membrane rafts as heterogeneous, dynamic nanodomains (10–200 nm) enriched in cholesterol and sphingolipids that can coalesce into larger platforms through lipid–protein and protein–protein interactions. Lipid rafts are small, dynamic nanodomains enriched in cholesterol and saturated sphingolipids that coexist with the surrounding liquid-disordered membrane due to the partial miscibility of lipid species. Their formation reflects the fluid–fluid phase separation driven by saturated acyl chains, extensive sphingolipid hydrogen bonding, and bulky glycosphingolipid headgroups, which collectively promote liquid-ordered regions stabilized by cholesterol [117,118]. Although highly transient and heterogeneous in living cells, these nanodomains create locally ordered environments that selectively recruit subsets of proteins and lipids, thereby shaping signaling pathways, membrane trafficking, and the spatial organization of the plasma membrane [119]. Although extensive work has since supported the existence and functional relevance of such short-lived nanoscale assemblies, their direct visualization in unperturbed living cells remains challenging, and no single model fully captures the complex physicochemical factors governing membrane heterogeneity.

Membranous vesicles (MVs) were first isolated by Taylor et al. in 1983 from the conditioned media of melanoma cells [120]. This early observation provided foundational evidence that cells release membrane-bound vesicles, initiating the modern field of extracellular vesicle (EV) research [121,122]. These lipid-bound structures are released by cells through various physiological mechanisms and are commonly classified as microvesicles, apoptotic bodies or exosomes. They are not simple fragments of the plasma membrane but rather highly organized nanodomains with unique lipid architecture. Small EVs, which are characterized by a lipid bilayer membrane, typically range in size from 100 to 200 nm and express common markers such as CD63, CD9, and Alix [123].

Exosomes specifically range from 30 to 150 nm and are distinguished by their specialized lipid composition, which includes SM, phospholipids, ganglioside GM3, and cholesterol. These lipids impart essential biophysical properties to the exosomes, including membrane curvature, rigidity, and stability [124]. Phospholipids, in particular, play a vital role as their remodeling leads to the generation of eicosanoids and other bioactive metabolites that can be implicated in cancer progression by promoting cell proliferation, enhancing angiogenesis, and inhibiting apoptosis [125]. The lipid composition of EVs is dynamic and can be influenced by external stimuli. For instance, metabolic precursors or inflammatory lipids have been shown to alter the lipid and protein cargo of exosomes, thereby affecting their biological activity. This flexibility emphasizes the potential of EVs as diseases biomarkers, such as in melanoma and their relevance in therapeutic contexts.

### 2.4. Methods for Investigating Membrane Lipid Structure and Dynamics

Membrane lipid composition and dynamics can be studied using a combination of biochemical, analytical, imaging, and biophysical approaches (see Table 1).

Lipidomics, typically performed using mass spectrometry/NMR-based platforms, allows for a comprehensive profiling of lipid classes and molecular species, providing quantitative information on lipid abundance, composition, and turnover. Stable isotope labeling further enables flux analyses, revealing rates of lipid synthesis, remodeling, and degradation [126,127]. Fluorescent lipid probes and dyes (e.g., BODIPY and Nile Red), including labeled phospholipids or cholesterol analogues, provide information on cellular distribution and composition. Notably, however, lipid probes and fluorescent dyes can alter the biophysical properties of lipids, potentially affecting their localization and trafficking. Although these probes remain essential for imaging lipids in cells, different studies highlight that the synthetic modifications required to generate bioorthogonal or environment-sensitive lipid analogues can alter the physical properties of native lipids, potentially affecting membrane organization and dynamics [128]. Live-cell imaging techniques, such as confocal microscopy, fluorescence resonance energy transfer (FRET) and super-resolution microscopy, reveal lipid localization, lateral organization, diffusion and dynamic interactions within the membrane [129].

Complementary biochemical fractionation techniques, such as detergent-resistant membrane isolation or density gradient centrifugation, enrich specific lipid domains for detailed molecular analysis. Model membrane systems, including liposomes, giant unilamellar vesicles (GUVs), and plasma membrane-derived vesicles, allow controlled experiments on lipid–lipid and lipid–protein interactions, membrane phase behavior, and the formation of liquid-ordered versus liquid-disordered domains. To probe lipid dynamics across multiple spatial and temporal scales, a variety of spectroscopic and scattering techniques are employed: nuclear magnetic resonance (NMR) and electron paramagnetic resonance (EPR) report on molecular motion and local lipid environment; fluorescence correlation spectroscopy (FCS) and dynamic light scattering (DLS) assess diffusion over micrometer length scales; X-ray photon correlation spectroscopy (XPCS) and neutron scattering methods, including small-angle neutron scattering (SANS), neutron spin echo (NSE), quasi-elastic neutron scattering (QENS), and inelastic neutron scattering (INS), provide non-invasive measurements of lipid motions from femtoseconds to hours and length scales from Angstroms to micrometers [130,131,132,133].

Molecular and genetic approaches provide mechanistic insights by modulating the expression or activity of specific enzymes, transporters, or regulators. Techniques such as gene knockout, knockdown (siRNA/shRNA), and overexpression allow researchers to probe the functional consequences of altered lipid metabolism, while reporter assays for lipid-sensitive transcription factors (e.g., SREBP, PPARs) monitor pathway activation [134,135]. Pharmacological interventions, using small-molecule inhibitors or activators, can selectively target lipid metabolic enzymes to assess their role in lipid composition, signaling, and cellular physiology [136].

Integrating these approaches provides a powerful framework to understand how membrane lipid composition and dynamics regulate essential cellular processes, including signaling, vesicle trafficking, membrane fusion, endocytosis, exocytosis, and the organization of membrane protein complexes. By combining quantitative, spatial, and temporal information, these methods enable a detailed understanding of the fundamental principles governing membrane structure, dynamics, and function [137,138].

## 3. Lipid Metabolism and Membrane Composition in Melanoma Behavior

### 3.1. Lipid Biosynthesis and Metabolic Plasticity in Melanoma

Lipid metabolism is profoundly reprogrammed in melanoma and influences the biophysical structure of the membrane, signaling, and tumor behavior. Melanoma cells thus increase their dependence on exogenous and newly synthesized lipids to support growth, survival, and metastatic dissemination [139,140]. An overview of the key pathways involved in lipid metabolism (uptake, synthesis, storage, and membrane remodeling) during melanoma progression is summarized in Table 2.

Many cancer types, including melanomas, can upregulate fatty acid transport proteins (FATPs), allowing tumor cells to import long-chain fatty acids from the microenvironment [157]. In addition, melanoma cells may increase de novo lipogenesis through the upregulation of FASN, the enzyme responsible for the conversion of acetyl-CoA and malonyl-CoA to long-chain fatty acids, which can be considered a reliable melanoma prognostic marker [158]. Sphingolipid metabolism is also dysregulated in melanoma (e.g., the general reduction in ceramide is frequently observed) and is associated with tumor progression and treatment resistance [159]. Interestingly, Bilal et al. demonstrated that sphingomyelin synthase 1 (SMS1), encoding by the gene *SGMS1*, or glucosylceramide synthase (GCS), encoded by the *UGCG* gene, were frequently downregulated in melanoma, leading to an enhancement of glucosylceramides instead of SM [147]. Furthermore, low SMS1 expression is associated with poorer prognosis in metastatic melanoma [147]. The importance of sphingolipid metabolism in melanoma progression is also underlined by other studies. Tang et al. demonstrated that CerS6 enzyme (ceramide synthase 6) expression was low in melanoma cell lines and that this expression correlated with the metastatic potential of these cell lines [151]. Moreover, acid ceramidase (encoding by *ASAH1* gene), which is responsible for the cleavage of ceramides into sphingosine and fatty acid, is highly expressed in melanocytes and proliferative melanoma cells in vitro and in patient biopsies [160]. Furthermore, Bizzozero et al. found that the expression of acid sphingomyelinase (ASMASE) was lower in primary melanomas than in benign nevi and was negatively correlated with melanoma aggressiveness [150]. Melanoma cells display also a strong dysregulation of glycosphingolipid metabolism. In particular, GD3 is considered a melanoma-specific antigen whose levels increase in melanoma cells compared to normal melanocytes and correlate with metastatic potential [161]. Moreover, several lines of evidence suggest that melanoma-derived gangliosides can contribute to an immunosuppressive microenvironment [162,163,164,165].

It is well known that sphingosine 1-phosphate (S1P), formed by the phosphorylation of sphingosine catalyzed by two isoenzymes (namely, sphingosine kinase1/2, SphK1/2), is a pleiotropic bioactive sphingolipid strongly implicated in cancer progression [166]. In detail, the SphK1–S1P axis is shown to drive melanoma progression by promoting tumor cell survival, shaping the microenvironment, and facilitating metastasis. Increasing the expression of SphK1 protects melanoma cells from ceramide-induced apoptosis [167], while its downregulation enhances antitumor immunity via M1 macrophage activation [168]. The SphK1–S1P axis also regulates crosstalk between melanoma cells and stromal fibroblasts [169], as well as melanoma and microglia in brain metastases [170], supporting highlighting SphK1 as a potential therapeutic target in melanoma progression. Therefore, it is clear that melanoma metastatic behavior is strongly influenced also by the lipid composition of the microenvironment [171].

Cholesterol biosynthesis and trafficking are frequently perturbed in melanoma, and recent studies highlight a link between metabolic alterations and melanoma progression. Changes in cellular sterol levels and in transport pathways influence membrane order, raft abundance and the localization/function of signaling receptors [172,173]. Elevated cholesterol and its metabolite 27-hydroxycholesterol (generated by the mitochondrial enzyme cholesterol 27-hydroxylase, CYP27A1) play a role in melanoma stem-like cell expansion, potentially reducing the efficacy of therapies such as vemurafenib (BRAFi) [174]. Interestingly, HDL cholesterol levels and dysglycemia are associated with increased melanoma metastasis, suggesting that systemic metabolic dysfunction may influence tumor aggressiveness and dissemination [175]. The importance of cholesterol homeostasis in melanoma has emerged from several studies and may be considered a potential therapeutical target. Kuzu et al. clearly demonstrated that leelamine (a chemotherapeutic agent that inhibits intracellular cholesterol transport) induces melanoma cell death by causing cholesterol accumulation, leading to autophagy inhibition [176]. Moreover, a prognostic signature of cholesterol synthesis genes has been associated with melanoma patient survival [148].

It is well established that melanoma is characterized by the consistent upregulation of key lipogenic genes, including *FASN* and *SCD*, which promote de novo fatty acid synthesis (from carbohydrates) and are associated with more aggressive phenotype and poorer prognosis [24,158]. Specifically, immunohistochemical analysis demonstrated that FASN expression is not only higher in malignant melanomas than in conventional benign nevi, it also increases with Breslow thickness, reaching the highest levels in metastatic melanomas [177]. These observations were confirmed also in oral melanoma [178]. It has been demonstrated that orlistat, a FASN inhibitor and a well-known anti-obesity drug, affects mouse B16-F10 melanoma cell growth both in vitro and in vivo [179]. Zecchin et al. further elucidated that orlistat and cerulenin (another FASN inhibitor) induce apoptosis in B16-F10 cells via the intrinsic pathway [180]. Moreover, orlistat impairs both metastasis formation and angiogenesis by reducing VEGFA (vascular endothelial growth factor A) [181]. FASN has been proposed also as potential target to overcome BRAFi resistance [182,183]. A recent study demonstrated that pharmacological FASN inhibition (using TVB-3664) in the presence of MAPK inhibitors results in increased sensitivity to ROS inducers, suggesting a combination of these therapies in melanoma [184]. Interestingly, patients with *FASN*-mutated melanoma also show a higher response rate to immune checkpoint inhibitors [185]. Saab et al. demonstrated through immunohistochemical analysis that along with FASN, ACC1 is also overexpressed in metastatic melanoma, whereas intracapsular nodal nevi were mostly negative, suggesting that both enzymes may be useful in identifying metastatic melanoma [186]. Finally, in a study of sun-induced melanoma, *FADS1* (which encodes fatty acid desaturase-1, also known as delta-5 desaturase) was reported to exhibit higher mRNA expression in melanoma tissue compared with normal skin [187].

Another lipid metabolism enzyme of particular interest is SCD1, whose expression is generally associated with poorer prognosis [188]. Interestingly, melanoma stem cells have been shown to survive treatment with BRAFi/MEKi by activating a metabolic resistance mechanism driven by SCD1 [145]. Enhanced SCD1 activity sustains melanoma stem cells viability by increasing YAP/TAZ transcriptional activity independently of ERK or AKT signaling. The pharmacological inhibition of SCD1 (using MF-438) selectively kills melanoma stem cells and partially restores sensitivity to BRAF/MEK inhibitors [145].

Lipid metabolic reprogramming is increasingly recognized as a central regulator also for immune escape in melanoma and resistance to immune checkpoint inhibitors [189]. In fact, it has been demonstrated that cholesterol accumulates in tumor-infiltrating CD8^+^ T cells, driving immune checkpoint expression and functional exhaustion [190]. Moreover, melanoma cell-derived glucosylceramide induced an ER stress response in tumor-associated macrophages (TAMs) by altering ER membrane lipid composition, leading to a pro-tumorigenic polarization of TAMs [191]. Interestingly, melanoma induces a local immune tolerance by reprogramming dendritic cells (DCs) via Wnt/β-catenin signaling, shifting them toward fatty acid oxidation [192]. There is also growing evidence that cancer-associated adipocytes (CAAs) become metabolically and functionally reprogrammed within the tumor microenvironment and contribute to tumor progression and immune response [193].

### 3.2. Linking Melanoma Genetics to Lipid Reprogramming and Therapeutic Interventions

In cutaneous melanoma, mutations in *BRAF*, *NRAS*, and *C-KIT* genes are frequent with the BRAF mutation being present in approximately 50% of cases, while the *BRAF* and *NRAS* mutations have to be considered mutually exclusive. *CDKN2A* is a tumor suppressor gene whose mutation is important in the hereditary predisposition associated with melanoma. Other mutations that may predispose to tumor syndromes are those occurring on *PTEN*, *TP53*, *BRCA1*, *BRCA2*, *RB1* genes. Therefore, mutations affecting such genes are of great prognostic relevance and important to address therapeutic choices with significant impacts on melanoma evolution and re-occurrence [194,195].

It is known that BRAF mutations account for approximately 50% of melanomas, which in the majority of cases occurs as a V600E type. The B-raf protein belongs to the Raf-kinase family involved in growth signal transduction through the regulation of the MAP kinase/ERKs signaling pathway whose key role in the regulation of secretion, cell division and differentiation phenomena is known. Therefore, inhibitors of these kinases are suitable drugs for the treatment of melanomas with BRAF mutations, representing the so-called target therapy [9]. Another gene whose mutation is frequently involved is NRAS, since this mutated gene is found in approximately 30–40% of melanomas arising on portions of skin that were continuously exposed to sun light even many years before the onset of the tumor. This mutation generally occurs as an alternative to those on the BRAF gene. The protein encoded by this gene is a GTPase of the RAS family [196]. The mutation affecting the C-KIT gene is among the least frequent; in fact, it is found in 1–3% of melanomas. However, it mainly occurs in tumors present on the palm of the hands, on the sole of the feet or under the nails (so-called acral freckles) or on the mucosal membranes. Sometimes, this mutation may be found in melanomas occurring on skin areas exposed to sunlight for a long time. The protein encoded by this gene is KIT (also known as CD117 or stem cell factor receptor), which is a transmembrane protein belonging to the receptor tyrosine kinase family [197]. Mutations within the CDKN2A gene can significantly increase the risk of developing melanoma but represent a hereditary predisposition factor also for other tumors such as pancreatic cancer. This gene encodes the p16 and p14ARF proteins whose function is to limit cell growth; therefore, their mutation can suppress such inhibitory functions, leading to a stimulatory effect on the proliferative rate of tumor cells [198]. Other genes like *PTEN*, *TP53*, *BRCA1*, *BRCA2* and *RB1* have been shown to be involved as hereditary predisposition factors for several tumors, including melanoma, although with a lower incidence than other neoplasms [199].

Regarding the involvement of the above-mentioned gene mutations on membrane lipids synthetic pathways, recent studies showed that cells expressing *BRAF* carrying the V600E mutation were enriched with immunomodulatory lipids like long-chain PUFAs, thus leading to the formation of long F-actin containing protrusions and lipid droplets accumulation [200]. In this study, also some plasmatic lipidomics evaluations were carried out, showing that long-chain fatty acids (e.g., SM, palmitic acid and adrenic acid) were increased, after the treatment, in plasma samples of patient non-responders to BRAFi/MAPKi drugs compared to controls. These findings suggest that BRAF mutation may address the metabolic flux toward a non-Warburg-like behavior. In this way, BRAF mutation plays a key role in the immunomodulatory lipid profile regulation [200], whose potential therapeutic implications have also been recently highlighted and reviewed [201]. Other studies showed how lipid metabolism may significantly affect melanoma sensitivity to BRAF inhibitors and influence melanoma cell response to the drugs [106].

It is should be highlighted that a direct involvement of the above-listed genes in the membrane lipids synthesis of melanoma cells and/or in the expression of lipid metabolism enzymes was not reported. On the other hand, recent studies showed that resistant melanomas to target therapy (BRAFi/MEKi-resistant) are characterized by lipid metabolism pathways downregulation, in particular through an altered expression of enzymes regulating lipid biosynthesis (e.g., FAs and cholesterol) [106,202]. Within the same studies, and others, the potential therapeutic effects of drugs active on membrane lipid biosynthesis pathways (in particular enzymes FASN and 24-dehydrocholesterol reductase) in combination with BRAFi were reported [106,182], suggesting a role of the BRAF-dependent pathway in these pathways. Similarly, other lipidogenetic mechanisms related to SREBP transcription factors were shown to mediate the resistance to BRAF-targeted therapy [183] as well as another study that demonstrated that SREBP1-regulated de novo fatty acid biosynthesis plays a key role in melanoma cells survival pathways [24]. On the other hand, it is noteworthy that the role of SREBP1 in cancer development and drug response has been reviewed [203].

Phospholipid peroxidation is considered a driving event leading to ferroptosis, which is a type of regulated cell death driven by the iron-dependent accumulation of lipid peroxides [204]; therefore, its targeting was proposed as a novel potential therapeutic approach in melanoma [205,206]. This is in line with other studies carried out through the analysis of primary and cultured circulating melanoma cells, in which authors demonstrated melanoma cell heterogeneity within lipogenic (through the SREBP action) and iron homeostatic pathways, suggesting that such heterogeneity can modulate the resistance to BRAF inhibitors and to ferroptosis-inducing drugs [25,207].

Regarding other genes whose mutations are less frequently found in melanoma patients, BRCA1 and BRCA2 are related either to ferroptosis resistance and to cancer-prone hereditary syndromes [208]; therefore, their mutations may play a role in melanoma cells’ membrane structure and stability by acting through ferroptotic-dependent phenomena. This is confirmed by the finding that lipid and metabolite deregulation was also found in the breast tissues of women carrying BRCA1 and BRCA2 mutations [209,210].

It should be also mentioned that PTEN-inactivating mutations are prevalent in melanoma and support tumor development though the hyperactivation of the AKT/mTOR pathway [9]. The PTEN-suppressive action plays a role in melanoma development and progression through its lipid phosphatase activity [211].

### 3.3. Membrane Lipid Alterations in Melanomagenesis and Metastatic Potential

**Figure 7:**
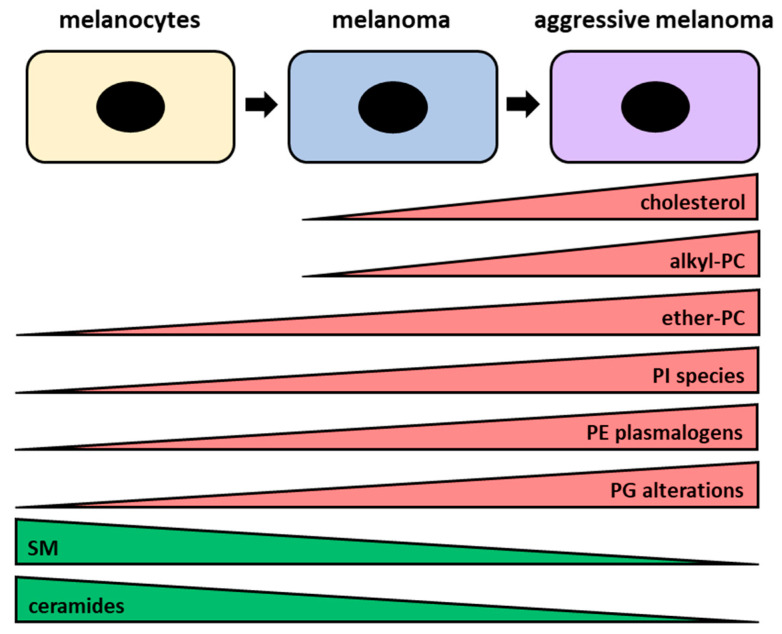
Schematic summary of membrane lipid remodeling during melanocytic transformation and melanoma progression. Melanoma progression and metastasis are strongly associated with alterations in membrane lipid composition. Aggressive melanoma cells show an increase in cholesterol and phospholipids as well as distinct lipid profiles compared with normal melanocytes. Lipidomic studies demonstrate that changes in ether-type phosphatidylcholines, sphingomyelins, certain plasmalogens, and phosphatidylinositol species can be considered potential indicators of melanoma malignancy.

It is well established that malignant cell lines display a distinct membrane fatty acid composition compared with their normal counterparts with a different fatty acid desaturation and incorporation that may underlie alterations in membrane structure and function [212]. Modifications in key lipids, including phosphatidylcholine, phosphatidylserine, and cholesterol, profoundly affect membrane organization, signal transduction, and metastatic potential [213] with melanoma being no exception [111]. In fact, early studies on B16 mouse melanoma cells reported differences in membrane organization, such as ganglioside and glycoprotein content, surface biochemical characteristics, and membrane lipid compositions associated with metastatic potential [214,215,216]. Moreover, other studies have highlighted the link between lipid composition and melanoma aggressiveness [217,218]. In human autologous melanoma cell lines with different biological characteristics, Le Bivic et al. demonstrated that highly tumorigenic cells exhibited an altered fatty acid profile, including increased free cholesterol-to-phospholipid and saturated-to-unsaturated fatty acid ratios, independent of pigmentation or tumor origin [217]. Similarly, it has been reported that highly metastatic B16-F10 mouse melanoma cells displayed an elevated alkyl-phosphatidylcholine compared to the less metastatic B16-F1 variant [218]. In recent years, lipidomic analysis has demonstrated that melanoma cells exhibit different membrane lipid alterations compared to melanocytes, which may reflect changes associated with malignant transformation and metastatic potential. Indeed, comparative analyses between melanocytes and melanoma cells, as well as between primary melanomas and metastatic cells, have revealed significant differences. To this aim, Perez-Valle et al., using an UHPLC-MS^E^ lipidomic approach, identified more than fifty significantly altered lipid species associated with malignant transformation [219]. In detail, compared with normal melanocytes, melanoma cells exhibited an accumulation of ether-type phosphatidylcholines (PCs) and a marked reduction in sphingomyelins (SMs). Moreover, melanoma cells showed an altered pattern of phosphatidylethanolamine (PE) plasmalogens and phosphatidylglycerols (PG) with two DHA-containing plasmalogens (PE P-16:0/22:6 and PE P-18:0/22:6) emerging as potential biomarkers of metastasis. Interestingly, distinct phosphatidylinositol (PI) and phosphatidylglycerol (PG) species were found to differentiate melanoma cells from normal melanocytes. In addition, phosphatidylinositol (PI) levels were higher in malignant melanoma compared with primary melanocytes, suggesting a lipid signature associated with melanoma progression [219]. Kim et al. previously reported similar findings, showing that specific phosphatidylinositol (PI) species progressively increase with metastatic potential, highlighting their potential as biomarkers [220]. The main differences in membrane composition are resumed in Figure 7.

**Figure 8:**
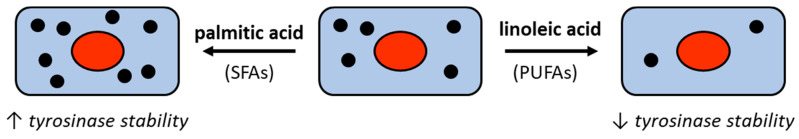
Simplified overview of how fatty acids and UV radiation influence melanogenesis and membrane remodeling in melanoma cells. In vitro studies on B16-F10 melanoma cells demonstrated that PUFAs suppress melanogenesis through multiple mechanisms, including the inhibition of tyrosinase (TYR) activity and promotion of TYR degradation. On the contrary, SFAs enhance melanogenesis by stabilizing TYR and promoting melanosome maturation.

As previously stated, melanin acts as a protective barrier by absorbing UV radiation in order to protect keratinocytes from DNA damages [221]. The synthesis of eumelanin and pheomelanin is initiated by tyrosinase (TYR), which is a copper-containing enzyme that catalyzes the initial steps of the pathway by converting tyrosine to DOPA (dihydroxyphenylalanine) and subsequently to DOPAquinone, after which the pathway diverges into distinct branches [8,222]. Melanogenesis occurs in melanosomes, which are lysosome-related organelles [223]. Interestingly, an early study demonstrated that eumelanosomes and pheomelanosomes differ in the quantitative, but not the qualitative, composition of phospholipids [224]. Furthermore, TYR transport into melanosomes is regulated by glycosphingolipid levels [225]. Fatty acids can modulate melanogenesis, regulating the stability of TYR [226]. Specifically, palmitic acid and eicosapentaenoic acid modulate melanin levels in melanoma cells by altering melanosome trafficking through the regulation of actin polymerization [226]. Moreover, PUFAs such as linoleic acid enhance TYR degradation via the ubiquitin–proteasome pathway, leading to a decrease in melanin production, whereas SFAs (like palmitic acid) stabilize TYR and increase melanin levels [227,228]. The effects of some FAs on TYR are reported in Figure 8.

**Figure 9:**
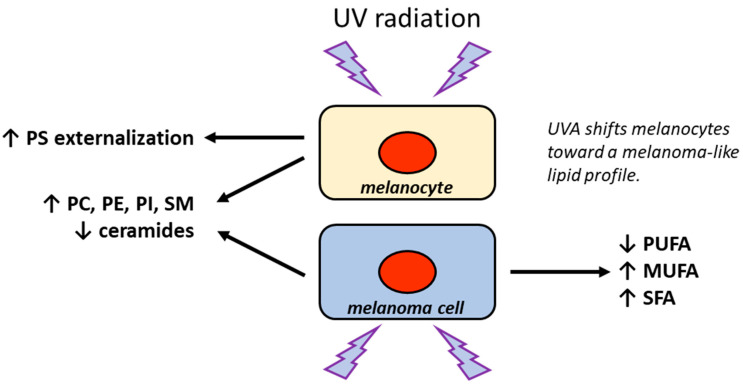
Schematic overview of UV radiation-induced membrane remodeling in melanocytes and melanoma cells. UVA exposure induces phospholipid remodeling in melanocytes that is similar to that observed in melanoma cells, which is characterized by increased levels of PE, PC, PI, and SM and a reduction in specific ceramide species. However, melanocytes exhibit a distinct response, notably the externalization of PS. In particular, high levels of PI are associated with the metastatic capacity of melanoma, suggesting that the induction of increased levels of this lipid following UV exposure may support a shift in melanocytes toward a melanoma-like phenotype.

As mentioned earlier, UV exposure plays a central role in melanoma pathogenesis through the induction of DNA damage, oxidative stress, and skin inflammation. In particular, UV radiation consists primarily of UVB (280–320 nm) and UVA (320–400 nm), which affect different layers of the skin. UVA penetrates deeper into the skin, reaching the dermis, and primarily causes indirect DNA damage via the generation of reactive oxygen species (ROS), resulting in single-strand breaks and DNA-protein crosslinks, while UVB (UVB is more cytotoxic and mutagenic than UVA) causes direct DNA damage through photoproducts formation [221,229]. UV exposure strongly affects membrane lipids due to the lipid peroxidation and the generation of ROS, which in turn oxidize polyunsaturated fatty acids, compromising membrane integrity and altering the organization of membrane microdomains. Interestingly, chronic UV exposure not only directly causes DNA damage but is also capable of reprogramming membrane composition and dynamics in keratinocytes [230]. The effects of UV exposure have been previously established in B16 melanoma cells. In fact, these cells respond to UV-induced oxidative stress through dynamic changes in lipid membrane (increase in ARA content and decrease in cholesterol/phospholipid ratio) [231]. Moreover, electron spin resonance analysis showed that UVB exposure rapidly induced lipid peroxidation and reduced membrane fluidity in B16 melanoma cells with ARA release from phospholipids [232]. A recent study demonstrated that UVA exposure induces several changes in the phospholipid content in melanocytes with respect to SK-MEL-5 melanoma cells. In both cell types, UVA radiation increases levels of phosphatidylethanolamine (PE), phosphatidylcholine (PC), phosphatidylinositol (PI), and sphingomyelin (SM) while reducing two ceramide species [233]. In contrast, phosphatidylserine (PS) content remained largely unaffected, but UVA radiation significantly increased the negative zeta potential of melanocytes, indicating PS externalization to the outer membrane leaflet [233]. It is well established that the externalization of PS is linked to apoptosis, immunosuppression, and melanoma development and metastasis [234,235,236]. Interestingly, the increase in PI levels in both melanocytes and melanoma cells after UVA exposure supports the idea that UVA may contribute to melanomagenesis [233], as this phospholipid is linked to the metastatic capacity of melanoma [219,220]. To better understand the effects of UV exposure, Maciel et al. investigated how UVA radiation alters the lipid composition of SK-MEL-28 melanoma cells using mass spectrometry-based lipidomics [237]. In detail, UVA exposure produced a decrease in polyunsaturated fatty acids (PUFAs) with a subsequent increase in saturated fatty acids (SFAs) and monounsaturated fatty acids (MUFAs), which is probably due to ROS-driven lipid peroxidation. Moreover, a reduction in PC levels and general increase in phosphatidylinositol (PI) has been observed [237]. The UV-mediated alterations in melanocyte and melanoma cell membrane composition are summarized in Figure 9.

As previously stated, cell membranes rapidly adapt to environmental stress by altering their lipid composition and microdomain organization. For example, altering the membrane fluidity of B16 melanoma cells using heat or chemicals can result in significant changes in lipid composition that promote lipid raft formation, such as the accumulation of cholesterol, ceramide, and saturated phosphoglycerides [238]. These results support earlier findings by Anderson et al., who demonstrated that heat-resistant melanoma variants exhibit characteristic shifts in membrane lipid composition, particularly in cholesterol levels [239]. It is well known that microenvironmental stress contributes to melanoma plasticity by shifting their phenotypes [240,241]. For example, low extracellular pH offers several advantages to melanoma cells [242] and may determine some membrane adaptations in cancer cells [243]. In particular, Mel501 melanoma cells grown in acidic conditions (pH 6.5) shift the phospholipid composition toward longer polyunsaturated free fatty acid and accumulate high levels of phosphatidylinositol (PI) among other phospholipids [243]. Therefore, the increase in PI can be considered a well-established lipidomic response observed in melanoma cells subjected to stress challenges (i.e., low pH, heat stress, and UVA exposure) [233,237,243], and it reflects mechanisms associated with melanoma aggressiveness [220].

Increased membrane fluidity, typically due to elevated levels of unsaturated lipids, increases cell membrane flexibility, thereby promoting the migration and invasion of cancer cells through extracellular matrix [244]. Generally, lipid composition alterations affect the formation and stability of lipid rafts (specialized membrane microdomains enriched in cholesterol and sphingolipids) that serve as organizing centers for signaling molecules [245]. Accordingly, in melanoma cells, cholesterol depletion via methyl-β-ciclodextrin disrupts raft organization, inhibits V-ATPase activity, and alters H^+^ efflux, impairing tumor migration and invasiveness [246], corroborating previous results on the use of methyl-β-ciclodextrin as a potential antitumor agent [247]. It is well established that an altered raft composition in melanoma cells enhances the activation of oncogenic pathways such as RAS/RAF/MEK/ERK, AKT/mTOR, and FAK/Src, promoting proliferation, survival, and epithelial-to-mesenchymal transition (EMT) [16,159,248,249]. Early evidence demonstrated that malignant melanoma cells exhibit enlarged lipid rafts that facilitate an aberrant activation of NFAT (nuclear factors of activated T cells), which is a key regulator of cancer cell growth and survival [249]. Interestingly, lipid rafts influence A375 melanoma cell motility by regulating lamellipodia formation via the actin-mediated recruitment of β1 and β3 integrins, promoting invasiveness [250]. Therefore, the lipid-metabolism-dependent modulation of membrane microdomains is a broader hallmark of melanoma cell signaling and represents a potential therapeutic target [28]. For example, persister cells with the BRAF(V600E) mutation survive BRAF/MEK inhibition through ACOX1 (acyl-CoA oxidase 1)-mediated fatty acid oxidation, which supplies energy and reduces oxidative stress, enabling survival under drug pressure [251]. Interestingly, xanthohumol, a natural flavonoid from hop (*Humulus lupulus* L.), enhances vemurafenib (a BRAFi) effects on SK-MEL-28 melanoma cells by depleting cholesterol and increasing membrane fluidity, facilitating drug uptake and sensitization [252]. At the same time, cholesterol-rich lipid rafts organize key signaling proteins, sustaining MAPK and PI3K/AKT pathway activity and further promoting therapy tolerance [253,254]. Moreover, Wu et al. demonstrated that the ABCA1 transporter, a key regulator of cholesterol and phospholipid efflux, regulates the plasma membrane organization of human melanoma cells by elevating cholesterol levels in order to enhance motility and aggressiveness [255].

### 3.4. Nutritional Modulation of Membrane Fatty Acids and Its Impact on Melanoma Progression

Fatty acids are key regulators of cancer lipid metabolism, with their availability and composition influenced by dietary intake and the tumor microenvironment, since MUFAs are fully synthesized endogenously, while humans are unable to produce PUFAs de novo. Cancer cells rewire metabolism to promote their growth, also increasing the uptake of external fatty acids [20]. To date, it is well established that beneficial health effects are achieved by reducing SFAs and substituting them with PUFAs to reduce the risk of metabolic disorders [256]. However, the so-called Western diet is typically rich in omega-6 PUFAs (high omega-6/omega-3 ratio), which can promote inflammation [257]. Moreover, omega-3 PUFAs are generally recognized for their anticancer properties [258]. Dietary PUFAs significantly influence the composition of membrane phospholipids. PUFAs, mainly at the sn-2 position of membrane phospholipids, can be hydrolyzed by PLA2s as free fatty acids, and then another fatty acid is incorporated into the phospholipid through a re-acylation reaction mediated by lysophospholipid acyltransferases (LPCATs). This deacylation/reacylation cycle, known as the Lands cycle, remodels membranes, regulates PUFA signaling, and maintains membrane stability [259]. Therefore, maintaining a balanced intake of omega-6 and omega-3 PUFAs is crucial, because the proportion of ARA (omega-6) versus EPA and DHA (omega-3) incorporated into membrane phospholipids determines whether PLA2 activation primarily produces pro-inflammatory eicosanoids (e.g., leukotriene B_4_, LTB_4_; prostaglandin E_2_, PGE_2_) or anti-inflammatory and pro-resolving mediators derived from EPA and DHA [20,31,260,261].

In melanoma, several lines of evidence highlight the potential role of PUFA-induced membrane remodeling in influencing and subsequently modifying tumor behavior. As an example, in a B16-F10 lung-metastasis mouse model, algal oil (rich in DHA) supplementation increased omega-3 PUFA levels in metastatic tissue and markedly reduced melanoma pulmonary metastasis. This effect was linked to the induction of autophagy, inhibition of mTOR and p38-MAPK signaling, activation of JNKs, and decreased secretion of pro-inflammatory cytokine interleukin-1β (IL-1β) [262]. Using the same mouse in vivo model, it has been demonstrated that only the oral administration of a 1:1 mixture of fish oil (enriched in omega-3) and soybean oil (enriched in omega-6) reduced tumor growth as well as pro-inflammatory mediators including LTB_4_, PGE_2_, and CXCL1 [263]. In another study on Fat-1 transgenic mice, which endogenously convert omega-6 to omega-3 PUFAs (since they express the *C. elegans fat-1* gene encoding a desaturase absent in mammals), it has been shown that the pulmonary metastasis of melanoma B16-F0 cells and inflammatory markers (i.e., chemokine receptor CXCR-4) are reduced compared with wild-type mice [264]. Moreover, the enrichment of PUFA content into membrane enhances melanoma susceptibility to oxidative stress and ferroptosis, as already reported in the previous paragraph [184]. Interestingly, among the ferroptosis-related genes considered, Chen et al. found nine risk genes that may influence melanoma clinical outcome, including genes related to lipid metabolism such as *ACSL4* (acyl-CoA synthetase long-chain family member 4), *ALOX5* (arachidonate 5-lipoxygenase), and *ACACA* (acetyl-CoA carboxylase alpha) [265]. In addition, PLA2G6 is markedly upregulated in melanoma tissue compared to normal nevi, and its silencing significantly inhibits cell proliferation and invasion while also reducing melanoma growth in vivo. At the same time, PLA2G6 may exert its effects through ferroptosis-related pathways [266].

### 3.5. Lipid Remodeling and Exosome Dynamics in Melanoma Biomarker Development

In addition to their structural and energetic functions, emerging evidence suggests that alterations in bioactive lipids, including plasma lipids and exosomes, could be used as prognostic and predictive biomarkers in various types of cancer. In melanoma, in particular, several lipid signatures have been detected in the plasma of patients [247]. Studies have also reported an enrichment of sphingolipids, including dihydroceramides, ceramides, SMs, hexosylceramides, lactosylceramides and GM3 gangliosides, in melanoma patients [152,153]. These findings suggest that these lipids may be useful for predicting treatment response. Higher levels of palmitic acid (FA 16:0), stearic acid (FA 18:0) and oleic acid (FA 18:1), as well as enhanced sphingolipid abundance, correlate with favorable therapeutic outcomes [267,268]. This further emphasizes the significance of this metabolic pathway in modulating drug sensitivity. Metabolic rewiring involving eicosanoid biosynthesis also contributes to therapeutic resistance. The increased synthesis of prostaglandin E_2_ (PGE_2_) and elevated oxidation rates of fatty acid have been associated with a poor prognosis in patients undergoing targeted therapies, such as BRAF and MEK inhibitors [269].

In addition to the observed alterations in circulating lipids, the lipid composition of extracellular vesicles, particularly exosomes, has emerged as a promising new area for the discovery of non-invasive biomarkers. Although exosomal lipidomics is still in its early stages in the context of melanoma, preliminary studies suggest that the lipid content of these vesicles reflects the metabolic state of the tumor cells from which they originate [267]. Melanoma-derived exosomes actively participate in tumor progression, immune modulation, and metastatic dissemination. Studies have shown that ganglioside-enriched exosomes, particularly those containing GD2, can promote melanoma proliferation, invasion, and adhesion by transmitting pro-tumorigenic signals to less aggressive cells [270]. Furthermore, melanoma-derived exosomes often contain high levels of sphingomyelin, lysoPC, phosphatidic acid and *bis*(monoacylglycero) phosphate, contributing to structural stability and endosomal dynamics [34].

Comparative analyses of exosomes released by melanoma cell lines with different invasive capacities suggest that these lipid signatures could be used to distinguish tumors with high metastatic potential from those with lower potential [271]. Tumor-derived vesicles containing prostaglandins and free fatty acids, including ARA, can modulate the tumor microenvironment, thereby fostering inflammation and promote conditions that are favorable to tumor growth and immune evasion [267]. Furthermore, melanoma patients consistently demonstrate elevated plasma concentrations of exosomes compared to healthy individuals, which is indicative of the high secretion rate of vesicles typical of melanoma cells. These vesicles often carry melanoma-associated antigens, immunosuppressive factors, metabolic enzymes, and distinct lipid species, highlighting their potential as a valuable source for “liquid biopsies” [267]. New avenues for therapeutic innovation are emerging, including strategies aimed at modulating membrane composition, targeting lipid biosynthetic pathways or interfering with exosome biogenesis. Together, these advances position lipid-centered diagnostics and therapeutics as promising additions to the landscape of personalized medicine in melanoma.

## 4. Conclusions

Melanoma remains the most aggressive form of skin cancer, which is characterized by early dissemination, therapy resistance, and a poor survival rate, especially in patients with metastatic disease. Melanoma cells depend on aerobic glycolysis for energy (Warburg effect) but maintain mitochondrial flexibility for adapting to hypoxia and nutrient stress [272]. This metabolic reprogramming at the mitochondrial level involves the β-oxidation of fatty acids and the catabolism of amino acids, whose products sustain the tricarboxylic acid cycle (TCA) and therefore oxidative phosphorylation (OXPHOS) [273]. In this scenario, lipid metabolism plays a particularly important role, as reported in this review. Key lipogenic enzymes (e.g., FASN and SCD1) are well studied in melanoma, where they are frequently upregulated and associated with proliferation, survival, and resistance to ferroptosis [145,200,274]. In contrast, data on these enzymes in normal melanocytes are limited, and much of the available evidence is based on comparisons between melanoma and melanocytic nevi, which are benign lesions that often harbor oncogenic mutations [275]. Consequently, the differences observed between melanoma and nevi cannot be directly interpreted as reflecting pathways active in normal melanocytes. Notably, Naimy et al. clearly highlighted that FASN and ACSL3 (acyl-CoA synthetase long-chain family member 3) show a low expression in nevi compared with the other melanoma subtypes [276]. Similarly, Lumaquin-Yin et al. demonstrated through a single-cell RNA sequencing approach that genes involved in melanogenesis and lipid oxidative metabolism have a coordinated regulation [277]. In fact, lipid droplet biogenesis in melanoma is regulated by DGAT1 (diacylglycerol O-acyltransferase 1). The upregulation of this enzyme enhances the lipid storage capacity of melanocytic melanoma, supporting tumor growth [277]. It is therefore clear that integrating multi-omics data is essential to highlight the complexity of cancer metabolism. This integrated approach would allow for a broader understanding of tumor heterogeneity, offering additional strategies to improve prognosis and personalize treatments [278,279].

In addition to addressing the role of lipid metabolism in melanoma transformation and aggressiveness, in this review, we also described how a diet-dependent enrichment of membrane PUFA content sensitizes melanoma cells to oxidative stress and ferroptosis, revealing metabolic vulnerabilities with therapeutic potential. Although some in vitro and in vivo studies demonstrate that omega-3 PUFAs may have beneficial effects against melanoma growth and progression, this topic requires further investigation, especially regarding the effects of omega-6 PUFAs [280]. Indeed, Mendelian randomization analyses using GWAS (genome-wide association study) data for melanoma and PUFA-associated genetic variants support this claim [281,282]. To our knowledge, only one case-control study has determined that the weekly consumption of fish rich in omega-3 fatty acids (in addition to other dietary factors present in the Mediterranean diet) may have a protective effect against cutaneous melanoma [283]. Therefore, systematic clinical investigations integrating dietary assessment, lipidomics, and functional analyses are needed to establish the translational relevance of these pathways.

Furthermore, published evidence on a large retrospective cohort showing that the “statin users” (i.e., people taking one of the most used classes of hypocholesterolemia drugs) showed a better overall survival when compared with not-statins users [284]. The clinical significance of this study is partially hampered by the composition of the enrolled cohort (veterans), which determines a relative lack of female subjects, but also by another more recent published study whose authors did not find a statistically significant association between statin use and sentinel lymph node metastasis by biopsy or melanoma recurrence [285]. Such controversial evidence may be due to the broad definition of “statin users” and also to the significant pharmacological differences among the components of this class of drugs. Further, due to the pleiotropic effects of these drugs [286], the mechanisms of action playing a role within these clinical studies remain to be identified and deserve more defined and better designed clinical studies. On the other hand, a central regulatory role of lipids in the immune escape and drug resistance of melanoma as well as the ability of lipid metabolism to regulate the of immunotherapy efficacy via ferroptosis in melanoma have been recently reviewed [189,287].

Finally, our review provided an overview on lipid signatures as promising biomarkers for melanoma. In fact, both the circulating lipid profiles and exosome structure differentiate melanoma patients from healthy individuals and correlate with therapeutic responses [247,250,251]. However, the clinical translation of these biomarkers is currently limited by the lack of standardized protocols for exosome isolation and the restricted availability of analytical platforms capable of accurately identifying and quantifying lipid species.

Overall, the available evidence supports the high potential of investigating lipid homeostasis to better understand cancer cell behavior, interactions with the tumor microenvironment, and signaling dynamics, ultimately enabling the development of novel prognostic and therapeutic strategies for melanoma patients.

## Figures and Tables

**Table 1:** Overview of multiscale approaches for studying membrane lipid composition and dynamics, listing the main techniques and their primary objectives.

Approach/Technique	Goal/What It Measures
Mass spectrometry-based lipidomics (LC-MS, HILIC-MS, ion mobility-MS)	Comprehensive profiling of lipid species, quantification, and lipid flux; analysis of synthesis, remodeling, and degradation
Stable isotope labeling	Tracks lipid metabolic fluxes and turnover rates
Fluorescent lipid dyes/analogs	Visualization of lipid localization, trafficking, lateral diffusion, and domain formation in live or fixed cells
Biochemical fractionation (e.g., detergent-resistant membranes, density gradients)	Isolation and enrichment of lipid microdomains (rafts) for compositional analysis
Model membrane systems (liposomes, GUVs, GPMVs)	Controlled studies of lipid–lipid and lipid–protein interactions, phase behavior, and domain formation
Nuclear magnetic resonance (NMR)	Local lipid motions, headgroup dynamics, and membrane fluidity at atomic resolution
Electron paramagnetic resonance (EPR)	Lipid packing, membrane order, and dynamics of spin-labeled lipids
Fluorescence correlation spectroscopy (FCS)	Measurement of lipid and protein diffusion and domain mobility on micron length scales
Dynamic light scattering (DLS)	Global lipid vesicle size and diffusion properties; membrane fluidity indirectly
X-ray photon correlation spectroscopy (XPCS)	Collective lipid motions and membrane dynamics at nanometer-micrometer scales
Neutron scattering techniques (SANS, NSE, QENS, INS)	Multiscale lipid dynamics: lateral diffusion, flip–flop, membrane bending, thickness fluctuations, phonon-like motions; time scales from femtoseconds to hours, length scales from Å to micrometers
Genetic manipulation (knockout, knockdown, overexpression)	Functional analysis of lipid metabolic enzymes or transporters; effects on membrane composition and organization
Pharmacological modulation	Study of specific lipid enzymes or pathways and their effect on membrane structure and dynamics

Abbreviations: small-angle neutron scattering, SANS; neutron spin echo, NSE; quasi-elastic neutron scattering, QENS; inelastic neutron scattering, INS.

**Table 2:** Changes in key enzymes involved in lipid metabolism during melanoma progression.

Pathways	Enzymes	Ex Vivo and Experimental Models	Expression	Outcome	References
Uptake	CD36	Metastasis-initiating melanoma cells	↑	Poor prognosis	[141]
	FABP7	Melanoma cells	↑	Proliferation, invasiveness	[142]
Biosynthesis	ACLY	Melanoma cells and in vivo models	↑	Poor prognosis	[143]
	ACSL3	Human database	↑	Poor prognosis	[144]
	FASN	Melanoma cells and patients	↑	Poor prognosis	[24]
	SCD1	Melanoma cells and patients	↑	Disease progression	[145]
	SPHK1	Melanoma cells and patients	↑	Shorter survival	[146]
	SMS1	Melanoma cells and patients	↓	Poor prognosis	[147]
	Cholesterol synthesis and import	Human database	↑	Poor prognosis	[148]
Storage	MAGL	Melanoma cells	↑	Aggressiveness	[149]
Membrane remodeling	ASMASE	Melanoma cells and patients	↓	Disease progression	[150]
	CerS6	Melanoma cells	↓	Aggressiveness of Cancer Cells	[151]
	Ganglioside metabolism	Melanoma cells and patients	↑	Disease progression	[152,153]
	PI3K	Melanoma cells	↑	Disease progression	[154]
	COX-2	Melanoma cells and patients	↑	Poor prognosis	[155]
	PTGES	Melanoma cells and patients	↑	Poor prognosis	[156]

Abbreviations: fatty acid binding protein 7, FABP7; ATP citrate lyase, ACLY; acyl-CoA synthetase long-chain family member 3, ACSL3; fatty acid synthase, FASN; stearoyl-CoA desaturase 1, SCD1; sphingomyelin kinase 1, SPHK1; sphingomyelin synthase 1, SMS1; monoglyceride lipase, MAGL; acid sphingomyelinase, ASMASE; ceramide synthase 6, CerS6; phosphatidylinositol 3-kinase, PI3K; cyclooxygenase-2, COX-2; prostaglandin E synthase, PTGES.

## Data Availability

No new data were created or analyzed in this study. Data sharing is not applicable to this article.
